# New Insights into SARS-CoV-2 and Cancer Cross-Talk: Does a Novel Oncogenesis Driver Emerge?

**DOI:** 10.3390/vaccines10101607

**Published:** 2022-09-25

**Authors:** Vasiliki Rapti, Thomas Tsaganos, Ioannis A. Vathiotis, Nikolaos K. Syrigos, Peifeng Li, Garyfallia Poulakou

**Affiliations:** 1 3rd Department of Internal Medicine, National and Kapodistrian University of Athens, 11527 Athens, Greece or; 2 1st Department of Internal Medicine, Alexandra General Hospital, 11528 Athens, Greece; 3 Harvard School of Public Health, Boston, MA 02115, USA; 4 Institute for Translational Medicine, Qingdao University, Qingdao 266021, China

**Keywords:** SARS-CoV-2, long-COVID, post-acute COVID-19 sequelae, post-acute sequelae of SARS-CoV-2, COVID-19 sequelae, cancer, oncogenesis, tumorgenesis, cancer progression, oncogenic pathways

## Abstract

Since the pandemic’s onset, a growing population of individuals has recovered from SARS-CoV-2 infection and its long-term effects in some of the convalescents are gradually being reported. Although the precise etiopathogenesis of post-acute COVID-19 sequelae (PACS) remains elusive, the mainly accepted rationale is that SARS-CoV-2 exerts long-lasting immunomodulatory effects, promotes chronic low-grade inflammation, and causes irreversible tissue damage. So far, several viruses have been causally linked to human oncogenesis, whereas chronic inflammation and immune escape are thought to be the leading oncogenic mechanisms. Excessive cytokine release, impaired T-cell responses, aberrant activation of regulatory signaling pathways (e.g., JAK-STAT, MAPK, NF-kB), and tissue damage, hallmarks of COVID-19 disease course, are also present in the tumor microenvironment. Therefore, the intersection of COVID-19 and cancer is partially recognized and the long-term effects of the virus on oncogenesis and cancer progression have not been explored yet. Herein, we present an up-to-date review of the current literature regarding COVID-19 and cancer cross-talk, as well as the oncogenic pathways stimulated by SARS-CoV-2.

## 1. Introduction

Coronavirus disease 2019 (COVID-19) is an infectious disease caused by severe acute respiratory syndrome coronavirus 2 (SARS-CoV-2), a novel coronavirus that emerged in the city of Wuhan, China, at the end of 2019. Unlike SARS and Middle East respiratory syndrome (MERS) outbreaks that temporarily caused considerable global health consternation, SARS-CoV-2 continues to pose unprecedented challenges for healthcare systems and socioeconomic structures worldwide, since its precise pathophysiology is still poorly understood [1,2]. Lately, research has been focusing on the identification of the long-term consequences of COVID-19 on human health and the quality of life and its impact on pre-existing or emerging diseases, including cancer [3,4,5].

As expected, the systemic immunosuppressive status of cancer patients, whether caused by the disease itself or the anticancer treatment, confers an increased risk of COVID-19 compared to the general population [6,7,8]. Several studies point out that cancer patients experience a longer and severer COVID-19 disease course and higher mortality rates are also observed [6,9,10,11]. Notably, cancer type, staging, and therapeutics are determinants of SARS-CoV-2-associated morbidity and mortality [9,12,13,14].

COVID-19 and cancer present clinical and molecular similarities. Immune dysregulation, manifested either as aberrant cytokine release or impaired adaptive immune responses, tissue hypoxia and damage, oxidative stress, and signaling pathways manipulation have been recognized as key components of both oncogenesis and COVID-19 etiopathogenesis [6,7,15]. Although cancer rarely results from an isolated event, the aforementioned eventually raise concerns about SARS-CoV-2 potentiality to behave as an oncogenic virus, predispose or accelerate malignant transformation and tumor progression [4,5,6,7]. The present review aims to summarize current knowledge on the overlapping mechanisms and the molecular interplay between SARS-CoV-2 and cancer and highlight virus ability to reshape the human cancer landscape.

## 2. Viral Infections and Cancer

Viral infections are known to drive oncogenesis and account for an estimated 15 to 20% of cancer cases worldwide [16]. So far, seven human viruses have been implicated in oncogenesis, namely Epstein–Barr virus (EBV), hepatitis B virus (HBV), human T-lymphotropic virus 1 (HTLV-1), human papillomaviruses (HPVs), hepatitis C virus (HCV), human herpesvirus 8 (HHV-8) and Merkel cell polyomavirus (MCPyV) [16].

Viral oncology has been a major research focus and a plethora of mechanisms and pathways contributing to oncogenesis have been reported. [16,17,18]. Despite the diverse genomes and cellular tropisms, oncoviruses share common characteristics that allow them to act either as primary etiological agents behind certain cancer types or as accumulating factors that over a long period of time and in conjunction with environmental and host-cooperating events predispose the human body to cancer development and accelerate its progression [16,17]. Like other viruses, they induce epigenetic changes and usurp host signal transduction pathways associated with cell-cycle regulation and metabolism, in order to optimize the cellular conditions for viral replication and immune escape [16,17,19]. Hence, they convey cancer hallmarks on the host cells by triggering unlimited cell proliferation and replicative immortality, inhibiting apoptosis, exploiting the DNA damage response network (DDR), evading immune responses, stimulating tumor-intrinsic inflammation, and promoting angiogenesis, invasion, and metastasis [18,20].

Oncoviruses encode viral proteins that target tumor suppressors’ pathways and activate cellular oncoproteins [21,22]. The function of p53 and the retinoblastoma protein (pRB) pathway, which tightly regulate cell cycle progression and induce apoptosis, is perturbed by viral oncoproteins [21,22]. For instance, oncoproteins encoded by HPV and EBV can suppress the activity of both p53 and pRB and drive cells into the S phase of DNA synthesis, permitting viral access to host replication machinery. Likewise, HTLV-1 oncoproteins transactivator from X-gene region (Tax), basic zipper factor (HBZ) and HBV-encoded hepatitis B X-antigen (HBx) oncoproteins inactivate p53 and interrupt p53-mediated apoptosis [16,21,22]. Loss of p53 or/and pRB-induced apoptotic signaling permits viral proliferation and spread before host cell death, thus manipulating antiviral immune responses and establishing chronic, persistent inflammation [23]. All the above eventually lead to genomic instability and accumulation of mutations predisposing to oncogenesis [24]. Other host signaling pathways subverted by oncoproteins, which impel cells towards malignant transformation, include phosphoinositide-3 kinase–protein kinase–mammalian target of rapamycin (PI3K–AKT–mTOR), mitogen-activated protein kinase (MAPK), Janus kinase/signal transducers and activators of transcription (JAK/STAT), Notch, nuclear factor kappa B (NF-kB) and vascular endothelial growth factor (VEGF) signaling [16,17].

Induction of oxidative stress and interplay with DDR network are two other mechanisms employed by oncoviruses granting genomic instability. Persistent viral infection triggers chronic inflammatory responses and reactive oxygen species (ROS) production. For example, EBV nuclear antigen (EBNA)-1 upregulates the transcription of nicotinamide adenine dinucleotide phosphate hydrogen (NADPH) oxidase resulting in ROS production, which, in turn, induces chromosomal aberrations and DNA double-strand breaks, and contributes to EBV-associated malignancy [25]. Oncoviruses are also capable of engaging DDR machinery to their advantage and establish a highly proliferative state [16]. The aforementioned gradually cause loss of genome integrity and mutations raise [26].

Furthermore, viral genome integration and epigenetic alterations have been linked to oncoviruses-mediated cancer development [17,19]. Both DNA and RNA viruses insert their viral genome into host cell DNA causing insertional mutagenesis and viral persistence in cells. The transformed cell enters an immortal state and acquires limitless replicative potential [21]. Simultaneously, insertional mutagenesis harbors tumor suppressor gene disruption and oncogenes overexpression [27]. Lastly, in order to regulate their life cycle, oncoviruses wield cellular gene expression through host DNA methylation, histone modification, chromatin remodeling, and viral-encoded non-coding RNAs (e.g., microRNAs, long non-coding RNAs, circular RNAs) and provoke host cell genome alterations [19,28].

## 3. Chronic Inflammation and Cancer

Chronic inflammation is considered to be one of the cardinal characteristics of tumor genesis, promotion, and progression, whereas it favors immune escape by suppressing immune surveillance and promoting the development of a tumor microenvironment (TME) that sustains cancer [29,30,31]. The potential link between inflammation and cancer was firstly proposed by Rudolf Virchow in the mid-19th century, based on the presence of inflammatory cells in tumor biopsies and the observation that tumors emerge at the sites of chronic inflammation [32]. Evidence suggests that tumor-related inflammation endows cancer cells with endless proliferative potential, resistance to growth inhibition and escape from programmed cell death, and enables angiogenesis, invasion, and metastasis [15]. Simultaneously, cancer treatment (e.g., surgery, chemotherapy, radiotherapy) can stimulate inflammatory responses and provoke tissue damage that may result in tumor recurrence and drug resistance [29].

Inflammation underlies a wide variety of processes essential for host defense against pathogens, tissue repair adaption to stress, and restoration of a homeostatic state [33]. In this context, acute inflammation has been proposed to enhance cancer immunosurveillance; it promotes the recruitment and the function of dendritic cells (DCs), which, in turn, stimulate anti-tumor inflammatory responses via diverse mechanisms, including cross-presenting tumor-associated antigens to cytotoxic T lymphocytes (CTLs) and recruitment of natural killer (NK) cells that sustain T-responses [34]. In case that acute inflammatory responses do not resolve properly or in time, chronic inflammation develops as a sequela. Within a dysregulated and persistent inflammatory state, cancer cells are able to subvert DCs function and a myriad of immunosuppressive cells (e.g., myeloid-derived suppressor cells [MDSCs], regulatory T cells [T_regs_], regulatory B cells [B_regs_], M2 tumor-associated macrophages [M2-TAMs], N2 tumor-associated neutrophils [N2-TANs] and T helpers 2 [Th2 cells]) is activated by cytokines, chemokines and inflammatory mediators secreted by tumor cells [29,31,35,36,37,38]. All the above eventually lead to oncogenes activation alongside tumor suppressor gene inactivation, induction of oxidative stress and DNA damage, stimulation of signaling pathways (e.g., NF-kB, K-RAS, etc.), and epigenetic alterations that reinforce cancer development [29,35,36].

### Cytokine Release

In chronically inflamed tissue, upon exposure to a plethora of inflammatory mediators, tumor cells become capable of responding to these signals, thus acquiring a growth advantage [39]. So far, several cytokines have been suggested to play a pivotal role in oncogenesis [36,39]. Transforming growth factor-beta (TGF-β), a highly pleiotropic cytokine that mainly regulates wound healing and angiogenesis, and prevents uncontrolled inflammation progression, is present in TME and enhances tumor cell invasion and metastasis [40,41,42]. Tumor necrosis factor-alpha (TNF-a), a pro-inflammatory cytokine that exerts various effects (e.g., apoptosis, necrosis, angiogenesis, immune cell activation, differentiation, and cell migration) depending on the cellular context, represents a double-edged sword in tumor initiation and development, since both pro- and anti-tumoral activity has been reported [43]. Tumor necrosis factor receptor (TNFR) activation leads to the stimulation of multiple signal transduction pathways, including NF-kB signaling that generates negative regulators of apoptosis (e.g., caspase-8 (FLICE)-like inhibitory protein [FLIPL], B cell lymphoma 2 [Bcl-2], superoxide dismutase) [44,45].

Although interleukin signaling holds a key role in tumor-directed immune responses, upon an oncogenic event, it can nurture an environment that harbors cancer growth [46,47]. In brief, persistent and aberrant inflammatory responses to pathogen-associated molecular patterns (PAMPs) trigger NF-kB signaling cascade, thereby enabling inflammasomes activation and active interleukin-1β (IL-1β) release from fibroblasts, epithelial cells, DCs, monocytes and macrophages [48]; ΙL-1β activates VEGF and promotes oxidative stress and DNA damage via production of nitric oxide (ΝO) and ROS by the epithelial cells [46,49]. IL-1β alongside TGF-β regulate the differentiation of Th17 cells and IL-17 release. Under homeostatic conditions, IL-17 contributes to the wound-healing process by triggering NF-kΒ. However, during chronic inflammation, it may recruit and transactivate epidermal growth factor receptor (EGFR) in skin cells, permitting their expansion and migration, and provoke general tumor outgrowth [46,50]. Moreover, IL-33 expression has been found to be upregulated within TME; tumor-initiating cells facilitate IL-33 secretion, which consecutively recruits TAMs, MDSCs, and T_regs_, therefore establishing a highly suppressive milieu for immune responses and contributing to further tumor spread [51]. In malignant cells and/or cells bearing oncogenic mutations, interleukins, such as IL-6, IL-11, and IL-22, stimulate STAT3 signaling that upholds cell proliferation, survival, stemness, epithelial–mesenchymal transition (EMT), and migration [46]; excessive STAT3 activation driven by IL-6 and IL-11 overexpression combined with oncogenic driver mutations mediate the development of solid tumors (e.g., lung, pancreatic, colon and gastric cancer) [46]. Among cytokines correlated with inflammation-induced oncogenesis, IL-6 predominantly regulates all cancer hallmarks and can be present in different stages of cancer development from promotion to metastasis [39,44,49,52,53]. IL-6 favors angiogenesis and tumor vascularization via VEGF secretion, inhibits oxidative stress and DNA damage related to cancer treatment by stimulating antioxidant and anti-apoptotic pathways, and contributes to cancer-related cachexia [46,49,53]. Furthermore, PI3K–AKT–mTOR signaling induced by IL-1 and IL-6 enhances further IL-1 and IL-6 production through metabolism shift towards glycolysis and the suppression of oxidative phosphorylation, thus aggravating inflammation-related carcinogenesis [46,54,55].

## 4. Immune Escape and Cancer

Cancer immunoediting serves as a conceptual framework that integrates the immune system’s dual role in tumor suppression and promotion [56,57]. It is a dynamic, multivariable process, in which robust immunological responses are produced in response to diverse tumor stimuli [58,59,60]. Immune cells (e.g., CTLs, NK cells, NKT cells, γδ T cells, and DCs) translocate in tumor sites and secrete various cytotoxic effectors, such as anti-angiogenic interferon-gamma (IFN-γ) and IL-4, perforin and granzyme [59,60]. Simultaneously, CD8^+^ T cells, NKs, and DCs recognize and lyse stromal or/and tumor cells through multiple mechanisms, including major histocompatibility complex (MHC)-, perforin- and tumor-necrosis factor-related apoptosis-inducing ligand (TRAIL)-dependent manner [60]. However, tumor cells apply a variety of strategies in order to debilitate cancer immunosurveillance, of which immunoselection (the selection of non-immunogenic tumor-cell variants) and immunosubvertion (the active suppression of innate and adaptive cytolytic effectors) are the most well documented [60,61,62].

Mostly present during the early stages of carcinogenesis, immunoselection is linked to antigen-processing and presenting machinery loss and desensitization to immune effectors, such as IFNs [59,60,62]. For example, downregulation or loss of human leukocyte antigen (HLA) class I molecules expression, as seen in melanomas, lung, and epithelial-cell cancers, outstrips immunological restraints and harbors immune escape [60,63,64]. Simultaneously, tumor cells interfere with molecules related to antigen presentation, such as transporter associated with antigen processing-1 (TAP-1), low-molecular-mass protein-2 (LMP2), lipoma preferred partner 7 (LPP7), and tapasin, the expression of which is deregulated during cancer development, thereby evading elimination [60,62,65]. Moreover, blockage of the granzyme B/perforin pathway through overexpression of the serine-protease inhibitor PI9, mutation of death receptors, methylation of the gene encoded caspase-8, and upregulation of FLIP are other mechanisms employed by tumor cells towards inhibition of CTL-mediated target-cell lysis [60,66,67].

Tumor cells can orchestrate a highly immunosuppressive microenvironment and immunosubvertion is thought to accompany advancing tumor growth [60,61,62]. For instance, tumor-associated myeloid cells can suppress T-cell function by overproducing NO and displaying increased arginase-1 activity [68]. Additionally, cancer cells impede CTL-mediated toxicity by releasing immunosuppressive factors, such as IL-10, TGF-β, programmed death ligand 1 (PD-L1), and prostaglandin E2 (PGE2), which further reduce immunogenicity [59,62,69]. IL-10, derived by monocytes, M2 cells, Th2 cells, mast cells, T_regs_, MDSCs, and mesenchymal stem cells (MSCs), activates STAT3-mediated suppressors of cytokine signaling 3 (SOCS3) expression, which, in turn, inhibits Th1 cell proliferation and alters Th1/Th2 differentiation [59,70,71]. PD-L1, abundantly expressed in numerous human cancers, induces T cell exhaustion by stimulating PD-1 signaling [72,73]. Additionally, interdigitating DCs and tumor cells overexpress indoleamine 2,3-dioxygenase (IDO) that is known to prevent immune-mediated rejection of the tumor cells, block CD8^+^ T cells proliferation, and favor CD4^+^ apoptosis [74,75]. TAMs, CD4^+^ T cells, CD25^+^ T cells, T_regs_, and MDSCs are other tumor-infiltrating immunosuppressive cells that dominate in the TME and sustain tumor progression [59]. For example, M2-TAMs are angiogenesis promoters and CTL response suppressors and have been associated with poor prognosis in patients diagnosed with breast or ovarian cancer [76]. Similarly, MDSCs abolish adaptive immune responses through induction of T_reg_ activity and M2 differentiation, deactivation of local T cells by cytotoxic oxidizing molecules secretion, interference with T cell migration, and blockage of NKs activation [59,77,78]. Finally, within immunosuppressive TME, tumor-specific CD8^+^ T cells are activated at the stage of initiation of tumor growth and gradually lose their cytolytic function at the later stages, hence promoting progressive tolerance to the tumor [60,79]. Likewise, during tumor expansion, tumor-specific CD4^+^ T cells lose their anti-tumor activity, whereas an increase in the number of T_reg_ cells is observed [80,81].

## 5. Gut Microbiota and Cancer

Gut microbiota is comprised of a heterogeneous population of commensal microorganisms (e.g., bacteria, fungi, archaea, and viruses) that colonize the intestinal tract system [82]. It performs multiple functions, including nutrient abortion, toxin metabolism, host immune system regulation, and prevention of resident pathogens expansion, whereas it is dynamically changed by internal (e.g., age, stress), dietary or environmental factors, as well as by infectious agents or antibiotic consumption [82,83,84]. The gut microbial balance is essential for homeostasis maintenance and any deviation from it, a condition known as dysbiosis, can have detrimental consequences for human health [85]. For instance, impaired gut microbiota has been associated with a plethora of human pathologies, such as inflammatory bowel disease, obesity, diabetes mellitus type II, non-alcoholic liver disease, cardio-metabolic diseases, cancer, and malnutrition [85,86].

In the context of cancer, ample evidence suggests the duality in the role of gut microbiota, since it can be either tumor-promoter or tumor-suppressor [87,88]. In fact, the relationship between microbiome and cancer is more complex than assumed; the microbiome may influence cancer progression and, in turn, cancer development may cause microbiome alterations [89,90].

Production of putative oncometabolites, perturbation of DDR network, and immune system manipulation are the leading mechanisms mediated by impaired gut microbiota towards cancer genesis and progression [89,91,92]. Within the dysbiotic gut, pathogenic bacteria produce and release a wide variety of protein virulence factors, some of which are protein toxins, that have a pro-carcinogenetic activity; they either induce DNA single- and double-strand breaks within the host’s epithelial cells or regulate crucial cellular proliferative pathways, thereby promoting a transient cell cycle arrest [92,93,94,95]. Failure to properly respond to DNA damage favors genomic instability and acquisition of malignant traits in predisposed cells [92,94,96]. Moreover, dysbiotic gut bacteria interfere with the DDR network. For example, in the case of *S. flexneri* and *H. pylori*, the secretion of *Shigella* virulence effector VirA and cytotoxin-associated-gene A (CagA), respectively, subverts the tumor suppressor function of p53, enhancing mutations raise during the DNA damage response in the infected cells [97,98,99]. Several pathogenic bacteria, such as *H. pylori*, *B. fragilis*, and *E. faecalis*, are also able to generate oxidative stress and consequently drive cell-autonomous genetic mutation [100,101].

Lastly, an impaired gut microbiome can drive inflammation-induced cancer through inflammation enhancement or inhibition of host immune responses [102]. Dysbiosis promotes the transcription of chemokine C-C chemokine ligand 5 (CCL5), which recruits a variety of innate and adaptive immune cells in the intestine, thus inducing the local activation of the IL-6 pathway and resulting in intestinal epithelial cell reprogramming [91,103]. As a consequence of local inflammation, toll-like receptors (TLR_s_) are upregulated by lipopolysaccharide (LPS) and other microbial products and stimulate cytokine release and the activation of signaling pathways (e.g., NF-kB, JAK/STAT3, c-Jun N-terminal kinase [JNK]) that are known to fuel tumor growth [91,104]. Moreover, commensal bacteria and their bioproducts induce inflammasomes activation and inflammasome-mediated IL-18 secretion, which ensure intestinal tissue remodeling and gastrointestinal barrier maintenance. However, within dysbiotic gut, IL-18 production is reduced due to the deficiency of inflammasomes components, which, in turn, causes larger commensal bacteria penetration and inflammation exacerbation and triggers tumor formation [89]. Finally, gut bacteria can inhibit immune effectors that prevent tumorgenesis [89]. For example, the Fap2 protein encoded by *F. nucleatum* interacts with T cell immunoreceptor with immunoglobulin and ITM domain (TIGIT), an inhibitory receptor present on NKs and various T cells, and impedes the NK-mediated tumor cell cytotoxicity [105].

## 6. Cytokine Release and Long-COVID

Cytokines play a crucial role in the evolution of infections, both microbial and viral, and determine the duration and severity of infections. Particularly for COVID-19, the course and outcome of the disease are determined by viral (mutational) and host (e.g., age, sex, comorbidities, and immunological) factors [106,107]. In SARS-CoV-2 infection, the innate and adaptive immune systems are activated following binding of the virus to the angiotensin-converting enzyme 2 (ACE2) protein of alveolar epithelial cells and subsequent production and interaction of chemokines, colony-stimulating factors, interferons, interleukins, and TNF-a. As vascular permeability increases, COVID-19 is developed [108,109]. When the immune response is dysregulated, it contributes to disease pathogenesis, as in the case of a “cytokine storm” [110,111], or hypercytokinemia, which is characterized by (a) perpetuated activation of lymphocytes and macrophages causing immune dysregulation, (b) large secretions of cytokines caused by such perpetuated activation, and (c) overwhelming systemic inflammation and multi-organ failure with high mortality [112,113]. The most important inflammatory mediators released by immune cells during the “cytokine storm” are IFN-α, IFN-γ, IL-1β, IL-6, IL-12, IL-18, IL-33, TNF-a, and TGF-β and they are associated with different clinical features of COVID-19 [106,114]. Indeed, cytokine storms correlate with the severity and progression of COVID-19 and can result in serious complications such as acute respiratory distress syndrome (ARDS) and multiple organ failure, which are the leading causes of death from the disease [115,116].

Shortly after the beginning of the COVID-19 pandemic, a phenomenon known as “long-COVID,” or post-acute sequelae of SARS-CoV-2 (PASC), appeared to medical professionals [117,118,119,120,121,122,123]. While COVID-19 symptoms are resolved in 1–4 weeks in the majority of patients, some patients complain of symptoms lasting longer than 28 days. These symptoms include fatigue, shortness of breath, headache (or “brain fog”), loss of smell (anosmia), sleep disturbances, fevers, gastrointestinal symptoms, anxiety, and depression and can persist for months and range from mild to disabling [124]. Even though a study on patients with pneumonia secondary to COVID-19 infection suggests that persistent symptoms may be attributable to “biopsychosocial” effects of COVID-19 (mainly due to the absence of residual radiographic abnormalities) [125], a number of studies have disclosed abnormal cardiopulmonary exercise testing [126], and most studies attribute the range of PASC symptoms to a number of differing pathological traits of the virus [127].

In the study by Queiroz et al., among patients in the post-COVID-19 group, subjects with PASC had higher levels of IL-17 and IL-2 and lower levels of IL-10, IL-6, and IL-4 than subjects without sequelae (i.e., without PASC symptoms/signs) [128]. The fact that pro-inflammatory cytokines were increased in the PASC group underlines the association of PASC with residual inflammation and inflammatory organ damage [129,130]. The higher levels of IL-10 and IL-4 in patients not suffering from PASC suggest better control of the inflammatory process due to increased levels of anti-inflammatory cytokines in these individuals. Furthermore, the significantly higher levels of IL-17 and IL-2 and lower levels of IL-4 and IL-10 in individuals with PASC suggest a possible “molecular signature” for PASC characterized by a Th17 inflammatory profile with a reduced anti-inflammatory response mediated by IL-4 and IL-10. In addition, since cytokines are prevalent in circulation, they are present in many different organ systems, and this explains the variety of symptoms associated with PASC. Nonetheless, since SARS-CoV-2 shows tropism for the nervous system and neurological manifestations (e.g., anosmia, ageusia, headache, stroke, Guillain–Barré syndrome, seizure, and encephalopathy) are observed in the majority of patients with acute COVID-19, microvascular inflammation in cells of the nervous system during acute infection may trigger mild symptoms of the disease [131], which may persist even after the infection has resolved.

In the study by Schultheiss et al., PASC appeared in up to 60% of the patients up to 24 months post-infection; elevated levels of IL-1β, IL-6, and TNF-a were detected in those patients with their most probable source of secretion being the monocytes and macrophages of the lung [132]. Similar results were observed in the study by Peluso et al.: higher levels of IL-6 and IFN-γ-induced protein 10 (IP-10) during early recovery from COVID-19 were associated with subsequent development of PASC; in addition, among patients with PASC, IL-6 showed a trend towards from early to late recovery [133]. Similar findings were noted in the study by Acosta-Ampudia et al., where a pro-inflammatory state was observed in patients with PASC characterized by up-regulated IFN-α, TNF-a, granulocyte colony-stimulating factor (G-CSF), IL-17A, IL-6, IL-1β, and IL-13, whereas IP-10 was decreased [134]. In yet another study, several cytokines from interferon I and III classes were highly elevated and stayed up for over 8 months [135].

The differences in the results among the previous studies could be attributed to methodological differences, e.g., different time-points of cytokine determination, population size, and characteristics (e.g., country, severity of acute COVID-19). Nonetheless, the paramount finding is a persistent and diffuse proinflammatory state and a dysregulated cellular immune response, which is compatible with two of the currently most discussed hypotheses on the immune pathogenesis of PASC: (a) ongoing immune responses against persisting virus or viral antigens and/or (b) chronic reprogramming of immune cells [128,132,133,134,135].

## 7. T-Cell Response and Long-COVID

The immune system is broadly divided into the innate immune system and the adaptive immune system. The adaptive immune system is important for the control of most viral infections. The three fundamental components of the adaptive immune system are B cells (the source of antibodies), CD4^+^ T cells (they possess a range of helper and effector functionalities), and CD8^+^ T cells (they kill infected cells). Adaptive immune responses are slow due to the intrinsic requirement of selecting and expanding virus-specific cells from the large pools of naive B cells and T cells: they take 6–10 days after priming to generate sufficient cells to control a viral infection, due to the inherent time demands for extensive proliferation and differentiation of naive cells into effector cells. Once sufficient populations of effector T cells (Th cells and CTLs) and effector B cells (antibody-secreting cells, known as plasmablasts and plasma cells) have proliferated and differentiated, they often work together to rapidly and specifically clear infected cells and circulating virions [136]. In a SARS-CoV-2 infection, the virus is particularly effective at avoiding or delaying triggering intracellular innate immune responses associated with type I and type III IFNs [137,138,139,140]. Without those responses, the virus initially replicates unabated and the adaptive immune responses are delayed, so that an asymptomatic infection or clinically mild disease follows; this is because the T cell and antibody responses occur relatively quickly and control the infection. Whenever impaired and delayed type I and type III IFN responses occur, the virus replicates heavily in the upper respiratory tract and lungs (due to failure of adaptive immune response priming) and a very high risk of severe or fatal COVID-19 ensues [141,142,143,144,145].

In PASC patients, the following findings have been observed: increases in antigen-specific CD4^+^ T cell responses to the SARS-CoV-2 S protein, antigen-specific activation in the circulating T follicular helper cells, and populations of CD8^+^ T cells (mainly attributed to their effector subpopulation). All these data show a prolonged T cell response magnitude [146], which eventually leads to exhaustion of T cells, as indicated by the increased expression of exhaustion markers, namely PD-1-expressing T lymphocytes [147,148,149]. Interestingly, T cell exhaustion can be reversed and T cell function can be restored by PD-1 blockade, which in turn ex vivo increases the CD4^+^ T cell-mediated response to SARS-CoV-2 spike and nucleocapsid peptides [147]. Another feature of PASC is the lower and more rapidly waning N-specific CD8^+^ T cell responses (IFNγ-/CD107a+ and IFNγ+); this lower frequency of degranulating virus-specific CD8^+^ T cells in individuals with PASC could be attributed either to the decreased functional capacity of these cells or dysfunction of the immune response [150]. Another feature of PASC is the significantly increased level of T_regs_ (CD4^+^ CD25^+^ CD127 low): this can be explained in the light of the increase in the PD-1-expressing T lymphocytes, indicating failing attempts of the immune system to control the persistent immune response [149]. Towards a better understanding of the pathophysiology of PASC, it has been suggested that T-cell subsets exhibit different dynamics, depending on the severity of the initial infection and the time since then; in severe convalescents, there is a tendency towards an exhausted/senescent state of CD4^+^ and CD8^+^ T cells and perturbances in CD4^+^ T_regs_ 3 months post-infection, a remodeling that is clearly visible at 6 months post-infection. In addition, CD8^+^ T cells exhibit a high proportion of CD57^+^ terminal effector cells, together with a significant decrease in the naive cell population, augmented granzyme B and IFN-γ production, and unresolved inflammation 6 months post-infection. On the contrary, mild convalescents showed increased naive T_regs_, and decreased central memory and effector memory CD4^+^ T_reg_ subsets [151].

Apart from circulating T and B cells, there are tissue-resident memory cells that reside in the peripheral non-lymphoid tissue [152,153]; those cells provide immediate and superior immunity against viral reinfections [154]. However, dysregulated lung-resident T cell responses may cause chronic lung inflammation and fibrosis after respiratory viral infection [155,156]. In line with those findings, Cheon et al. have found that exuberant CD8^+^ T cell responses may be connected to the development of chronic lung sequelae after the resolution of acute COVID-19 infection in aged individuals [157].

## 8. Tissue Damage and Long-COVID

The recent coronavirus outbreaks (SARS epidemic of 2003 and MERS outbreak of 2012) have demonstrated that persistent respiratory symptoms and radiographic abnormalities continue beyond the period of acute illness. In a considerable number of these patients post viral sequelae in the form of lung fibrosis have been reported. Given the genetic homology between SARS-CoV-2 with both SARS and MERS and the increased infected population worldwide during the current pandemic, it can be assumed that an increased likelihood of post-COVID-19 long-term pulmonary complications, and particular lung fibrosis, is expected [158]. In COVID-19 survivors, more than one-third describe respiratory symptoms in the context of PASC; of those greater at risk are females, who had severe acute COVID-19 disease and/or required invasive or noninvasive ventilation.

Pulmonary fibrosis can occur in the setting of maladaptive resolution of lung injury or exaggeration of the reparative process [159]. Fibroblasts respond to alveolar injury and secrete the extracellular matrix; during acute infection and the post-COVID period they are overreactive due to the upregulation of inflammatory cytokines and this results in distortion and remodeling of the pulmonary architecture. Monocyte-derived alveolar macrophages (MoAMs) are profibrotic and stimulate and form reciprocal circuits with fibroblasts [160]; the maladaptive repair of the alveolar injury results in a positive feedback loop that results in filling the alveolar interstitium and alveoli with matrix proteins and fibroblasts. In this setting, PASC-pulmonary fibrosis may develop following a prolonged phenotype of a slowly unfolding, spatially limited inflammatory alveolitis [161]. The prolonged exposure of profibrotic monocytes to elevated levels of cytokines results in intense interaction with fibroblasts to promote fibrotic repair processes. Altogether there is concern that the protracted nature of acute COVID-19, persistence of high levels of cytokines, monocyte/macrophage and T-cell circuits stimulating potential circuits between monocytes and fibroblasts, coupled with the extended duration of mechanical ventilation, could combine to promote a milieu in the alveoli with an increased likelihood of PASC-pulmonary fibrosis [134]. SARS-CoV-2 can also impose lung tissue damage by impairing microcirculation with a number of mechanisms [162]: capillary pericytes are damaged in COVID-19, thus hindering lung repair and neo-angiogenesis; endothelial cells (EC) become apoptotic and protrude in the lumen; capillary endothelium glycocalyces’ are shed; capillaries are obstructed by circulating neutrophils, by microthrombi formation or by fibrin-rich amyloids formation which are resistant to fibrinolysis [163,164].

Blood microcirculation disturbances during COVID-19 and subsequent tissue damage that may contribute to PASC can also affect other organs [162]. In the heart, endothelial infection results in EC swelling in small vessels and scattered necrosis of individual myocytes. In the brain, infection of subcortical white matter microvessel endothelium is associated with hyper-acute, microscopic ischemic lesions, and older ischemic and hemorrhagic microscopic lesions. In the skin, EC infection is associated with endothelial swelling and in some patients with thrombosis and fibrinoid necrosis in surrounding tissue. Keeping in mind that erythrocyte diameters are bigger than the capillary lumen, endothelial damage is likely to disturb capillary flow patterns in all affected organs, resulting in extreme shunting of oxygenated blood through the shortest capillary pathways. Thus, it has been proposed that COVID-19 is an endothelial disease and PASC may have its origins in subsequent tissue hypoxia [162,165].

Tissue damage has been disclosed in many organs of convalescent people, even in young ones, mostly free of risk factors for severe COVID-19: more than half of them had at least one radiographic abnormality of the lungs, heart, liver, pancreas, kidneys, or spleen and these abnormalities could persist for at least 2–3 month after hospital discharge [166]. Although not a pure tissue damage, gut microbiome disruption (i.e., gut dysbiosis) has been observed among patients with COVID-19 and persists even after disease resolution. Gut dysbiosis is also correlated with increased COVID-19 severity and inflammatory biomarkers and prolonged SARS-CoV-2 [167]. It has also been postulated that the gut microbiome modulates the neurotransmitter circuitries in the gut and brain via the microbiota gut–brain axis.

## 9. Gut Microbiota and Long-COVID

COVID-19 is an infection that primarily affects the human respiratory tract. Nonetheless, around 5–33% of COVID-19 patients have gastrointestinal (GI) symptoms, including diarrhea, nausea, and vomiting [114,168,169], and SARS-CoV-2 has been detected in stool samples and anal swabs [170,171]. These findings are in line with the enhanced expression of ACE2 in the GI tract, rendering the digestive tract an extra-pulmonary site for SARS-CoV-2 infection [172]. Since the GI tract is the largest immune organ in humans, playing critical roles in combating infections of pathogens [173], it can be assumed that the trillions of microorganisms inside the gut of humans are regulating host immunity [174]. A number of studies have disclosed that the gut microbiome ecology is broadly altered in patients with COVID-19 and that the gut microbiome configurations are associated with immune responses and disease presentations in COVID-19, in both the short term and long term [167,175,176,177,178], which in return influence the human host’s health.

### 9.1. Compositional Changes of the Gut Bacterial Microbiome

In the study by Zuo T et al. [179], it was shown that during acute COVID-19 the gut bacterial microbiome of patients was characterized by significant depletion of beneficial commensals and enrichment of opportunistic pathogens in the gut compared with healthy controls [177]. Interestingly, these alterations in the bacterial microbiome ecology persisted even after the clearance of SARS-CoV-2 from the respiratory tract [177]. A similar pattern of gut microbiome dysbiosis in COVID-19 patients was disclosed in the studies by Tang et al. and Gu et al., where the (beneficial) butyrate-producing bacteria were significantly decreased in patients with COVID-19 with a corresponding abundance of the common opportunistic pathogens (e.g., *Enterobacteriaceae*, *Enterococcus*, *Streptococcus*, etc.) [180,181].

The human digestive tract is also affected in the long term by COVID-19, rendering it one of the systems affected during PASC [182]: prolonged shedding of viral RNA in stool specimens up to 42 days and the presence of SARS-CoV-2 virus in the gut epithelium up to 90 days after disease resolution [183,184]. Concordantly, long-lasting gut microbiome dysbiosis is also consistently observed in subjects who recovered from COVID-19 [167,177,185,186,187,188], implying that the gut microbiome is closely linked to host health in a post-COVID-19 age [189]. In a six-month follow-up study on the gut microbiome of patients with COVID-19, significant decreases in the richness of the gut microbiome were observed across the acute, convalescence, and post-convalescence phases of COVID-19 [185]. In addition, COVID-19 patients had a significantly reduced gut bacterial diversity [181,190]. It should be noted that microbial diversity is a critical determinant of microbial ecosystem stability [191] because stable ecosystems provide colonization resistance to opportunistic pathogens [192]. Therefore, the reduction in gut microbiota diversity and richness may somewhat contribute to the expansion of opportunistic bacteria and have a long-term impact on patients with COVID-19 [193], especially in the light of the persistence of certain perturbations even after disease resolution [177,181] and the presence of different PASC symptoms (including respiratory and neuropsychiatric symptoms) [194,195].

### 9.2. The Gut Mycobiome in COVID-19

The human gut also harbors a large number of fungi, known as the gut mycobiome. The gut fungi have been demonstrated to be causally implicated in microbiome assembly, ecology, and immune development [196,197]. In the study of Zuo et al., patients with COVID-19 also presented with alterations in the gut mycobiome, characterized by enrichment of *Candida albicans* and highly heterogeneous mycobiome configurations [176]. The abundance of opportunistic fungal pathogens, *Candida albicans*, *Candida auris*, and *Aspergillus lineages*, was increased in the feces of COVID-19 patients during the disease course [176,198]. Fungal pathogens associated with pneumonia and respiratory symptoms, *Aspergillus flavus* and *Aspergillus niger*, were detected in the fecal samples from a subset of patients with COVID-19, even after disease resolution [176]. Unstable gut mycobiomes and prolonged dysbiosis persisted in a significant proportion (~30%) of COVID-19 patients [176]. These data suggest a gut mycobiome dysbiosis in COVID-19 and its relationship with a systemic dysregulation of host immunity.

Overall, such fungal bloom in the gut of patients with COVID-19 is likely a result of SARS-CoV-2 infection. Secondary fungal infection or co-infection in patients with COVID-19 during the pandemic was frequently observed [199,200,201]. *Candida albicans* has been shown to impair gut microbiome assembly, including gut microbiome reassembly after disruption by antibiotics and inflammation [202,203]. This detrimental effect of *Candida albicans* and *Aspergillus lineages* in the microbiome reassembly has been demonstrated in a fecal microbiota transplantation (FMT) study in *Clostridium difficile* infection, where fungi impair colonization of donor bacteria into recipients [203]. These studies suggest a crucial role of gut fungi in the gut microbiome ecology, revealing the centrality of simple microbial–microbial interactions in shaping host-associated microbiota. Moreover, gut colonization by *Candida albicans* can aggravate inflammation in the gut and non-gut tissues [204,205]. Therefore, the opportunistic expansion of certain fungi in COVID-19 patients potentially has a deleterious role on gut microbiome assembly, where a persistent gut microbiome dysbiosis is consistently seen even after disease resolution and hospital discharge.

### 9.3. The Gut Virome in COVID-19

In addition to bacteria and fungi, the human gut also harbors an immense diversity of viruses collectively known as the gut virome [206,207]. The virome consists of both RNA and DNA viruses that chronically infect their eukaryotic (humans, animals, and plants) and prokaryotic hosts (bacteria) [207]. The gut virome serves to modulate the ecology of the co-resident gut bacterial microbiota as well as the immunity of the mammalian host [206].

Even in the absence of GI symptoms and after respiratory clearance of SARS-CoV-2, an active presence of SARS-CoV-2 in the fecal RNA virome was found in 47% of patients with COVID-19 [208]. Meanwhile, more eukaryotic viruses were enriched in the feces of COVID-19 patients [175], as a result of SARS-CoV-2 infection. The eukaryotic viruses may harness the immune dysfunction of the host after SARS-CoV-2 infection to expand [207]. Thus, the gut virome in COVID-19 showed more stress-, inflammation-, and virulence-associated gene coding capacities [175]. Phages associated with opportunistic bacteria were also enriched in the gut virome and their increase was proportional to the increase in their host bacteria as a result of SARS-CoV-2 infection [180,186]; the co-expansion of phage and their respective bacteria has also been reported in gut inflammation [209,210].

Gut inflammation per se is able to boost bacteriophage transfer between bacteria [211]. Therefore, the alterations in the ecology of gut virome, particularly in the bacteriophage community, are at least partly caused by the alterations of the bacterial microbiome under the influence of SARS-CoV-2 infection and the subsequent immune dysfunction. Similarly, the gut virome dysbiosis persisted along with the dysbiosis of the gut bacterial microbiome, even after disease resolution of COVID-19 [175]. Thus, the strong correlation between the composition of virome and bacterial microbiome observed in COVID-19 patients was no surprise [212].

Along with the phenotypic changes in the host in SARS-CoV-2 infection, the gut microbiome (consisting of the bacterial microbiome, mycobiome, and virome) is broadly altered in COVID-19. Moreover, subsequent blooms of opportunistic bacteria, fungi, and viruses under the circumstances of SARS-CoV-2 infection and quiescent/overt gut inflammation in COVID-19 result in decreases in microbiome diversity. In conjunction with the impaired host immunity, such alterations may hinder the reassembly of the gut microbiome post-COVID-19, resulting in a significant weakening of the intricate microbiome ecological network in a steady state and contributing to the development of PASC.

## 10. Oncogenic Pathways and SARS-CoV-2

**Figure 1:**
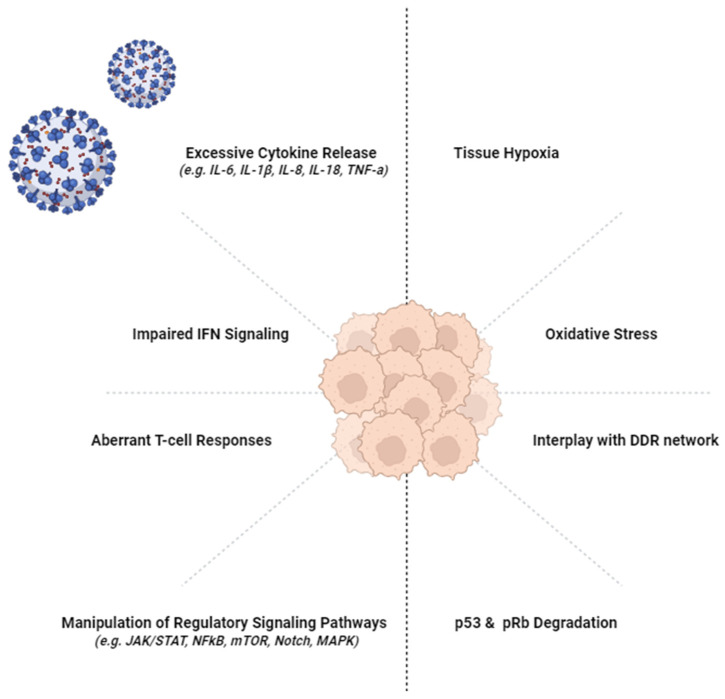
The shared molecular mechanisms between SARS-CoV-2 and oncogenesis. The figure was generated using images assembled from BioRender (available online: https://biorender.com, accessed on 30 August 2022).

Growing evidence suggests that SARS-CoV-2 can stimulate oncogenic pathways, hence raising concerns about its potentiality to reshape the human cancer landscape. Interplay with the DDR network, aberrant cytokine release, impaired T-cell responses, manipulation of regulatory signaling pathways, oxidative stress, and tissue hypoxia are the main mechanisms employed by SARS-CoV-2 that are suspected to contribute to carcinogenesis as a COVID-19 sequela (Figure 1) [213,214,215].

As discussed above, viruses interfere with the DDR network by recruiting DNA damage proteins to viral replication centers and regulating apoptosis via repair pathways suppression. As such, SARS-CoV-2 non-structural protein 1 (NSP1) has been shown to interact with all four members of DNA polymerase alpha (Pol a), an essential complex that is involved in the initiation of DNA replication and couples cycle cell progression to DRR [216]. Like other RNA viruses, in order to be replicated and escape immune surveillance, SARS-CoV-2 manipulates directly or indirectly mitochondrial metabolism, a key component of cellular homeostasis maintenance and cancer pathophysiology, by interfering with mitochondrial proteins [217]. Gordon et al. reported the interaction of SARS-CoV-2 ORF9c, a membrane-associated protein enabling immune evasion, with electron transport chain (ETC) components, suggesting a possible role as an oxidative phosphorylation regulator [216]. It is worth mentioning that ETC induces oxidative stress, as well. SARS-CoV-2 NSP8 and NSP5 have been proposed by Tutuncuoglou et al. to interact with histone methyltransferase NSD2 and HDAC2, respectively, and the latter have been linked to several oncogenic pathways [213,218]. For example, both of them are involved in NF-kB activation by proinflammatory cytokines, whereas NSD2 overexpression results in oncogenic RAS-driven transcription in lung cancer cells, and HDAC2 is a coactivator of the tumor suppressor p53 [219,220]. Lastly, the SARS-CoV NSP3 and NSP15 have been implicated in the degradation of p53 and pRb, respectively, and the SARS-CoV-2 S2 subunit has been demonstrated in silico to interact with p53 and BRCA 1/2, granting genomic instability [221,222,223]

Immune dysregulation, a hallmark of COVID-19 course and severity, is mainly orchestrated by cytokines and chemokines (e.g., IL-6, IL-1β, IL-8, IL-18, TNF-a) that are identified as tumorigenesis drivers [35]. Additionally, multiple signaling pathways (e.g., IL-6/JAK/STAT, NF-kΒ, IFN-Ι), which are prominent in oncogenesis, have been proved to contribute to COVID-19 etiopathogenesis, as well [214]. For instance, aberrant IL-6/JAK/STAT-3 signaling potentiates inflammatory responses in COVID-19, therefore aggravating disease severity; in the context of cancer, it attenuates anti-tumor T-cell mediated responses and favors tumor growth, survival, invasiveness, and or/metastasis [224]. Furthermore, the NF-kB pathway has been documented to be hyper-activated in moderately or critically ill COVID-19 patients and it is characterized as a crosstalk mediator between inflammation and cancer [225]. The NF-kB pathway is capable of remodeling the immune landscape to benefit tumor proliferation, inhibiting apoptosis and attracting angiogenesis [225]. Impaired IFN signaling, which underlies severe COVID-19, employs pro-tumoral properties and it is assumed as a key mechanism in tumor proliferation [226]. IFNs have been reported to trigger the NF-kB pathway, protect cells against apoptotic stimuli and foment angiogenesis [226,227,228]. Interestingly, IFNs harbor immunoevasion by decreasing sensitivity to NK cells and downregulating tumor-associated antigen presentation and contribute to drug resistance via IFN-related DNA damage-resistant signature (IRDS) induction [226,229].

It is no surprise that SARS-CoV-2 and tumor cells interfere with similar signaling pathways to their advantage. mTOR pathway displays fundamental functions (e.g., proliferation, metabolism, protein synthesis, autophagy, and apoptosis) and it is perturbated in cancer [230]. SARS-CoV-2 is also known to exploit mTOR signaling and at least seven targets have been identified so far [231,232]. The Notch pathway, a highly conserved signaling pathway that controls multiple cell differentiation processes, is an attractive target in COVID-19 and its overexpression enhances viral entry and the manifestation of inflammatory, coagulopathic and hypoxic events [233]. In addition, it is one of the most commonly activated signaling pathways in different cancer types and emerging data highlight its contribution to invasion, tumor heterogeneity, angiogenesis, or tumor cell dormancy within solid cancer tissues [234,235]. The p38 MAPK pathway allows cells to interpret and respond to various signals and stimuli, such as DNA damaging genotoxic agents, inflammatory cytokines, oxidative stress, and heat shock [236]. In COVID-19, it has been stated as disproportionately upregulated as a result of ACE2 activity loss upon viral entry or/and direct viral activation of p38 MAPK; the activated p38 MAPK pathway facilitates viral entry via ACE2 endocytosis and predisposes for pathological processes, such as inflammation and thrombosis [237]. Dysregulated p38 MAPK signaling is implicated in a wide range of cancers and it oversees the expression and the deposition of pro-angiogenic and pro-tumorigenic factors aiding in carcinoma growth, metastasis, and treatment resistance [236,238].

Persistent tissue hypoxia, derived by hyperinflammation or SARS-CoV-2-induced ACE2 depletion, promotes oxidative stress and genomic instability. Within predisposed cells, the proteomic and genomic changes may initiate cell cycle arrest and apoptosis evasion, while they facilitate tumor overgrowth, invasion, and metastasis [239,240]. In the case of SARS-CoV-2-mediated oxidative stress, NO overproduction alongside bradykinin degradation stimulates EGFR signaling, which is commonly upregulated in diverse carcinoma types [231].

## 11. Conclusions

Long COVID or PASC is an emerging condition that has attracted public interest, and scientists are gradually piecing together the puzzle of the lingering disorder that affects some of the convalescents. Although little is known about its precise etiopathogenesis, it seems that common pathogenic mechanisms intervene in PASC occurrence and oncogenesis, as depicted in Figure 1. So far, SARS-CoV-2 has not been proven to integrate into the host genome and act as a traditional oncovirus. Nevertheless, by exploiting host immunity and stimulating signaling and oncogenic pathways, it raises concerns about his ability to establish an oncogenic microenvironment. Simultaneously, since COVID-19 vaccination has been suggested to reduce the likelihood of PASC, of interest is its potential involvement in the suppression of the oncogenic pathways triggered by SARS-CoV-2. Future studies designed to further explore the intersection between SARS-CoV-2 and cancer and the disposition of the patients experiencing long-COVID for cancer are needed.

## Data Availability

Not applicable.
